# Supplementary material for: The development of the “Laab Nuer Model” for food safety management in handling traditional Lanna cuisine in Thailand
Source: PLoS One. 2025 Sep 26;20(9):e0331933. doi: 10.1371/journal.pone.0331933 (PMC12469109; doi:10.1371/journal.pone.0331933)
Supplement: S1 Table — (PDF) [file pone.0331933.s001.pdf]

Supplementary Material

S1 Table. Questionnaire for the owner or the food handler in the traditional Lanna restaurant

Direction Please give the X into the blank related to your agreement

Item	Your opinion	
	True	False
1. Unclean food can cause diarrhea.		
2. It is acceptable to wear rings while preparing food.		
3. Cooked food should be left at room temperature for a long time.		
4. Borax is commonly found in minced pork.		
5. Formalin is often found in certain types of seafood, such as squid and shellfish.		
6. Chili powder that appears whitish or moldy can still be used in cooking after heating.		
7. Ingredients should be chopped before washing them.		
8. Used cooking oil can be reused for frying more than three times.		
9. Baking soda cannot remove toxic residues from fruits.		
10. Proper handwashing requires all 7 steps and should take at least 20 seconds.		

Thank You!
