# Supplementary material for: The development of the “Laab Nuer Model” for food safety management in handling traditional Lanna cuisine in Thailand
Source: PLoS One. 2025 Sep 26;20(9):e0331933. doi: 10.1371/journal.pone.0331933 (PMC12469109; doi:10.1371/journal.pone.0331933)
Supplement: S2 Table — (PDF) [file pone.0331933.s002.pdf]

Supplementary Material

S2 Table. Questionnaire for the downstream

Direction Please give the X into the blank related to your agreement

Items	Level of agreement				
	**5 (Strongly agree)**	**4**	**3**	**2**	**1 (Disagree)**
1. Food safety management at the upstream level					
1.1 random checks of fresh market produce					
1.2 agriculture education to generate safe food					
1.3 purchasing safe farm products at above-market pricing					
2. Food safety management at the midstream level					
2.1 sampling from restaurants for contamination assessment					
2.2 organizing the local food safety education					
2.3 establishing a food safety committee					
3. Food safety management at the downstream level					
3.1 promoting food-safe establishments					
3.2 signs indicating a restaurant's safety and hygiene					
3.3 promoting food safety through fairs or exhibitions					
3.4 raising food safety awareness and knowledge among students and local teenagers					

Other comments or suggestions:

Thank You!
