# Supplementary figures and images for: The development of the “Laab Nuer Model” for food safety management in handling traditional Lanna cuisine in Thailand
Source: PLoS One. 2025 Sep 26;20(9):e0331933. doi: 10.1371/journal.pone.0331933 (PMC12469109; doi:10.1371/journal.pone.0331933)

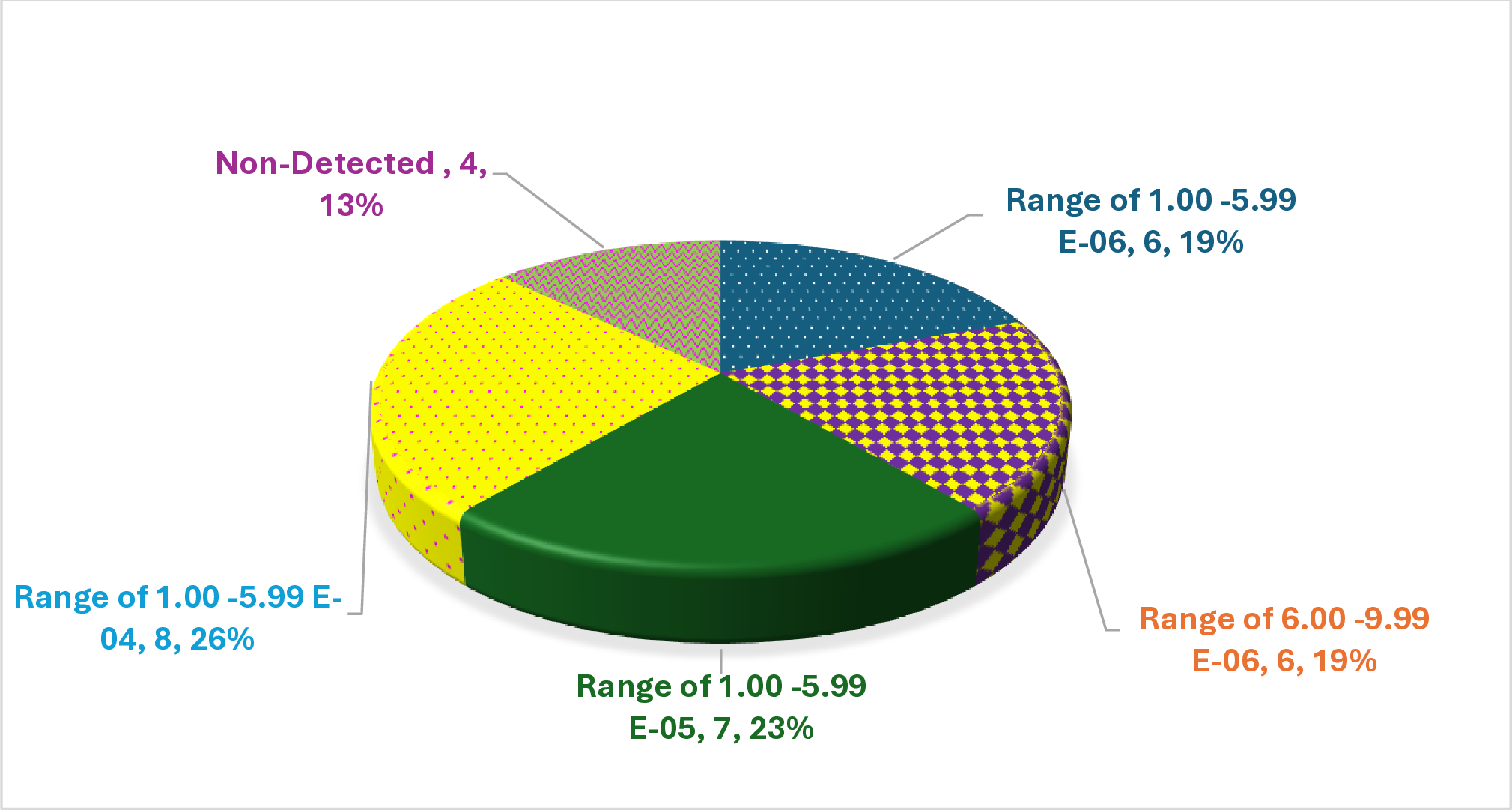

Supplement: S3 Fig — (TIF) [file pone.0331933.s003.tif]
