# Supplementary material for: The development of the “Laab Nuer Model” for food safety management in handling traditional Lanna cuisine in Thailand
Source: PLoS One. 2025 Sep 26;20(9):e0331933. doi: 10.1371/journal.pone.0331933 (PMC12469109; doi:10.1371/journal.pone.0331933)
Supplement: S4 Table — (PDF) [file pone.0331933.s004.pdf]

Supplementary Material

S4 Table. The statistical testing from the consumer from the IBM SPSS version 20.0

Item	Statistic Values (n=306)				
	**Average**	**Levene's test for homogeneity of variances**	**p-Value (From ANOVA test)**	**Eta Squared (η^2)**	**Affecting to the food safety management for traditional Lanna Cuisine (Yes/No)**
1. Food safety management at the upstream level					
1.1 random checks of fresh market produce	4.65	0.425[a]	0.00[b]	0.305[c]	Yes
1.2 agriculture education to generate safe food	4.58	0.143[a]	0.00[b]	0.334[c]	Yes
1.3 purchasing safe farm products at above-market pricing	4.28	0.404[a]	0.00[b]	0.334[c]	Yes
2. Food safety management at the midstream level					
2.1 sampling from restaurants for contamination assessment	4.65	0.209[a]	0.00[b]	0.368[c]	Yes
2.2 organizing the local food safety education	4.58	0.102[a]	0.00[b]	0.410[c]	Yes
2.3 establishing a food safety committee	4.59	0.207[a]	0.00[b]	0.443[c]	Yes
3. Food safety management at the downstream level					
3.1 promoting food-safe establishments	4.52	0.109[a]	0.00[b]	0.355[c]	Yes
3.2 signs indicating a restaurant's safety and hygiene	4.62	0.117[a]	0.00[b]	0.369[c]	Yes
3.3 promoting food safety through fairs or exhibitions	4.50	0.101[a]	0.00[b]	0.284[c]	Yes
3.4 raising food safety awareness and knowledge among students and local teenagers	4.58	0.166[a]	0.00[b]	0.331[c]	Yes

[a] all p-Value for Levene's test for homogeneity of variances had more than 0.05, indicated that the assumption of homogeneity was met

[b] It was compared with the 0.05 of significant level

[c] It was interpreted that this factor had a high effect according to the study of Richardson (2011)
