# Supplementary material for: The development of the “Laab Nuer Model” for food safety management in handling traditional Lanna cuisine in Thailand
Source: PLoS One. 2025 Sep 26;20(9):e0331933. doi: 10.1371/journal.pone.0331933 (PMC12469109; doi:10.1371/journal.pone.0331933)
Supplement: S5 Table — (PDF) [file pone.0331933.s005.pdf]

Supplementary Material

S5 Table. Summary of the findings, practical strategies, and responsible persons

Levels of operations	Summary of the findings		Practical strategies and Responsible persons
	**Strength**	**Weakness**	
1. Upstream operation (Fresh market)	- The chemical contamination permitted under Thai standards includes borax, salicylic acid, formalin, sodium hydrosulfide, and paraquat.<br><br>- it provides an initiative to promote the concept of good agricultural practices (GAP).	- It lacks support for the higher production costs if certain agriculturists have proof that their products are safe for consumers.	***Central government, local government, and the holder of fresh market should;***<br><br>- monitor the raw material in the market.<br>- provide or promote some of encourage some farmers to participate in projects or activities linked to appropriate agricultural practices.<br>- support the high value of production price if some agriculturist can claim that their products are safe to the customers.
2. Midstream operation (Traditional Lanna Restaurants)	- Food handlers and the holders of restaurants actively engage in enhancing their personal hygiene.	- Some food handlers have not successfully completed the training on proper personal hygiene.<br>- Some restaurants absence the assessment of food hazards and sanitation practices.	***Local government, the holder of restaurant, and food handler should;***<br><br>- monitor their raw materials and encourage for the relevant sector<br>- complete or pass the food safety training<br>- participate in food safety activities
3. Downstream operation (Customers)	- they acknowledge with these topics of food safety management for traditional Lanna cuisine from the customer sector;<br>1) informing consumers about food safety sources and restaurants<br>2) making signs to encourage food safety and sanitation in handling traditional Lanna cuisine<br>3) holding food safety exhibitions<br>4) providing food safety education to local students	- they lack an understanding on how to engage in food safety management within traditional Lanna cuisine	***Central and local government should;***<br><br>-promote a food safety promise to other customers<br>-receive training on food safety and sanitation for younger generation
