# Supplementary material for: The development of the “Laab Nuer Model” for food safety management in handling traditional Lanna cuisine in Thailand
Source: PLoS One. 2025 Sep 26;20(9):e0331933. doi: 10.1371/journal.pone.0331933 (PMC12469109; doi:10.1371/journal.pone.0331933)
Supplement: Attachment — Submitted filename: reviewer_report_malicakir.docx [file pone.0331933.s007.docx]

**Dear Editor,**

**Review of Manuscript PONE-D-24-37780: “The Development of the Laab Nuer Model for food safety management in handling traditional Lanna cuisine at Thailand”**

Thank you for the opportunity to review this manuscript, which presents a model designed to improve food safety in the preparation and handling of traditional Lanna cuisine in Chiang Rai, Thailand. The study explores upstream, midstream, and downstream aspects of food safety and proposes the “Laab Nuer Model” as a comprehensive solution. While the manuscript addresses an important public health issue and offers a locally grounded perspective, several areas require improvement in terms of clarity, methodology, and presentation.

### **Strengths of the Manuscript**

**Relevance and Originality:**
The manuscript addresses a critical public health concern—foodborne illnesses—in the context of local Thai cuisine. The development of a culturally rooted, multi-level food safety model is both innovative and relevant.

**Methodological Breadth:**
The division of operations into upstream (market), midstream (restaurants), and downstream (consumer) levels provides a holistic framework for food safety management.

**Practical Orientation:**
The proposed interventions—such as food safety training and consumer education—are realistic and applicable beyond the study context.

### ***Major Concerns***

#### **1. Language and Grammar**

The manuscript would benefit greatly from comprehensive language editing. There are multiple grammatical issues, awkward phrasing, and inconsistencies in terminology (e.g., "consume" instead of "consumer level"). These impede the overall readability and professionalism of the manuscript.

**Recommendation:** A native-level or professional academic English editor should revise the manuscript prior to publication.

#### **2. Clarity and Justification of the “Laab Nuer Model”**

While the concept of the model is innovative, its structure and rationale are not clearly described. There is a need for stronger theoretical and contextual grounding of each component.

- What makes the model unique compared to existing food safety interventions?
- Why is it called “Laab Nuer”? Is the model intended to generalize beyond that specific dish?

**Recommendation:** Include a conceptual framework early in the paper and explain the name, components, and theoretical basis of the model.

#### **3. Methodological Issues**

**Upstream:**
The use of HPLC for Paraquat analysis is appropriate, but the explanation of sampling strategy is unclear. How were the 31 samples selected? Were they random?

**Midstream:**
The selection of 25 restaurants via purposive sampling lacks detailed justification. How were hygiene standards measured? What standards were applied to evaluate E. coli contamination?

**Downstream:**
Sample size calculation via G*Power is appropriate, but the questionnaire design, validation (IOC/Cronbach’s Alpha), and implementation need clearer explanation.

**Recommendation:** Include a more structured “Study Design” section under Methods. Justify sampling, elaborate on data collection tools, and include the actual questionnaire as supplementary material.

#### **4. Results Presentation**

- Figures and tables (e.g., Paraquat levels, E. coli before/after) lack clarity and appropriate labeling.
- Statistical data (ANOVA results) are presented but not interpreted in depth.
- Descriptive statistics are overemphasized; inferential analysis is underdeveloped.

**Recommendation:** Improve visual quality and labeling of figures. Consider including confidence intervals or effect sizes. Move raw data and long tables to supplementary material.

#### **5. Discussion and Implications**

The discussion would benefit from deeper engagement with the literature. While some local studies are cited, the discussion lacks comparison with global standards (e.g., HACCP, WHO five keys to safer food).

**Recommendation:** Expand the discussion to situate findings within international food safety frameworks and similar intervention models.

#### **6. Ethical Considerations**

The ethical approval is stated and appropriately included. However, details such as informed consent procedures should be mentioned explicitly.

### **Minor Concerns**

- APA 7 citation inconsistencies should be corrected.
- Clarify abbreviations (e.g., MJPK test-kit) at first use.
- Improve the visual design of conceptual figures (Figures 1, 5, 6).
- Some reference links are repeated or broken in the reference list.

### **Recommendations for Improvement**

1. **Language Editing:** Revise entire manuscript for grammar and clarity.
2. **Conceptual Model:** Provide a theoretical framework for the model.
3. **Methods Transparency:** Clarify sample selection and tool validation.
4. **Data Presentation:** Improve visual quality of figures/tables.
5. **Literature Integration:** Strengthen discussion with international comparisons.

**Conclusion**

The manuscript addresses an important issue in food safety through a culturally grounded and multi-layered approach. With improvements in writing, methodological transparency, and critical discussion, the “Laab Nuer Model” has the potential to make a valuable contribution to the literature on local food safety management practices.

Sincerely,

M. Ali ÇAKIR, *PhD, MSc*

Assistant Professor,

Kırklareli University,

Dept. of Nutrition and Dietetics

m.ali.cakir@klu.edu.tr
