# Supplementary material for: The development of the “Laab Nuer Model” for food safety management in handling traditional Lanna cuisine in Thailand
Source: PLoS One. 2025 Sep 26;20(9):e0331933. doi: 10.1371/journal.pone.0331933 (PMC12469109; doi:10.1371/journal.pone.0331933)
Supplement: Attachment — Submitted filename: 090825 Response to Reviewers_VK.doc [file pone.0331933.s011.doc]

**Reviewer #4**

**(Green Hi-light)**

**4.1 Language and Clarity**
Although minor language edits were applied throughout the manuscript, significant issues with grammar, sentence construction, and scientific clarity remain (e.g., use of terms such as “consume level,” “may be received knowledge”). These affect the intelligibility of the work.

**Recommendation:** Please seek professional English editing service to ensure clarity and fluency appropriate for international publication.

**The revision**

Thank you for your initial comments regarding the language and clarity of the manuscript. Based on your recommendation, we submitted the manuscript for professional proofreading by an experienced academic editor from the United Kingdom. This revised version has already been proofread by this professional editing service. In addition, the expert of English editing suggest that the name of article should be present as the entitle “**The Development of the “Laab Nuer Model” for food safety management in handling traditional Lanna cuisine in Thailand** ”

**4.2 Conceptual Framework of the Laab Nuer Model**

You have now defined the Laab Nuer Model in terms of upstream, midstream, and downstream components, and included a conceptual diagram. However, it remains unclear why this specific dish (“Laab Nuer”) was chosen to model general food safety behavior. The cultural justification and theoretical rationale are still insufficiently developed.

**Recommendation:** Elaborate on the theoretical foundation of the model and its potential for broader application. Discuss how this model aligns or differs from internationally recognized frameworks such as HACCP or WHO guidelines. Strengthen conceptual integration in both Methods and Discussion sections.

**The revision:**

From this valuable recommendation, I revise this information as list follows;

- **Expanded the explanation** of how *Laab Nuer* represents a culturally embedded, high-risk traditional dish with symbolic importance and widespread consumption in Northern Thailand. (Line 110-122)
- **Described the model’s structure** using the upstream–midstream–downstream framework to mirror the food safety risks and practices across the supply chain, in line with the farm-to-fork approaches. (Line 123-127)
- **Clarified alignment with international standards**, including HACCP principles and WHO food safety guidelines, while emphasizing how the model integrates local practices and informal food systems. (Line 129-135)
- **Strengthened conceptual coherence** across the Methods and Discussion sections by explicitly connecting the Laab Nuer Model with the European Commission's farm-to-fork strategy and the WHO's guidance on safe and culturally appropriate food systems. (Line 508-519, 520-525)

**4.3 Methodological Transparency**

Improvements have been made in explaining the use of purposive sampling, and some methodological details have been added (e.g., IOC, Cronbach's alpha). However: The criteria for selecting restaurants using purposive sampling remain vague. The actual questionnaire remains inaccessible, and response items are not described. The data collection instruments, and their validation still lack detailed documentation.

**Recommendation:** Include clear selection criteria for participants and settings. Provide the questionnaire as supplementary material. Describe how validity and reliability were assessed.

**The revision**

Following the reviewer’s recommendation, we have added the inclusion criteria for the downstream study in lines 224-228. A purposive sample size diagram for traditional Lanna restaurants has also been constructed and is now presented in Fig. 3 at the page of 7. In addition, the questionnaire used for the customer survey has been provided as Supplementary Material B (see line 260). Furthermore, we have explained the procedures for content validity and reliability assessment, including IOC and Cronbach’s alpha analysis, in lines 272-282.

**4.4 Statistical Reporting**

The application of one-way ANOVA is noted, but statistical assumptions (normality, homoscedasticity) are not evaluated. Also, effect sizes or confidence intervals are not reported, reducing the robustness of the interpretation.

**Recommendation**: Explicitly test and report assumptions for ANOVA. Include effect sizes (e.g., eta-squared) and interpret findings accordingly.

**The revision**

From the valuable recommendation, we add the calculation of normality by the Kolmogorov–Smirnov test (line 286-287), and we report the Levene’s test for homogeneity of variances as can be seen in the Supplementary Material D. The effect size of the ANOVA analysis has been added up in the methodology and the result. For the effect size interpretation, we select the effect size interpretation by the study of Richardson (2011), which indicated that an effect size greater than 0.14 is a large effect (Line 284-285, 367-368).

**4.5 Discussion and International Context**

The manuscript continues to emphasize local observations without adequately situating them within international food safety literature. Global guidelines (FAO/WHO, HACCP) are minimally referenced and not critically compared to the study’s findings.

**Recommendation:** Integrate relevant international food safety studies. Discuss how your findings align with or diverge from established global practices.

**The revision**

Thank you for the recommendation. We have changed the discussion section to make it clear how the Laab Nure Model corresponds with international food safety standards, such as the World Health Organization (WHO) guidelines, the Codex Alimentarius standards, the One Health concept, and the HACCP framework. While the Laab Nure Model does not emphasize formal documentation or regulatory compliance in the same way as HACCP, it instead focuses on enhancing local knowledge and awareness of food safety within the context of traditional cuisine. These revisions can be found in lines 508-532 of the revised manuscript.

**4.6 Ethics and Data Availability**

While ethical approval is mentioned and verbal informed consent is reported, there is no justification for why written consent was not obtained. The PLOS ONE data sharing policy also appears insufficiently addressed.

**Recommendation:** Justify the use of verbal rather than written consent. Clearly state where and how the underlying data are available or justify any restrictions.

**The revision:**

We apologize for this mistake on the ethics and data availability. In fact, we use the written informed consent form for all participants of this study that was approved by the Chiang Rai Provincial Health Office (experimental protocol no. CRPPHO 131/2564). Moreover, we mention that all participants have written the informed consent form for this study, as can be seen in lines 168-169.

**Reviewer #5**

**(Yellow Hi-light)**

5.1 *As I cannot see whether the authors have comprehensively addressed the previous reviewer’s comments, I have a question about this revised version: what is the rationale for determining this sample size?*

**The revision:**

Thak you for your valuable recommendation. From this address, we can explain the reason of sample size collection as lists follows;

1)    The study area is mentioned at Chiang Rai, Thailand, because this area is the origin of traditional Lanna Cuisine related to the study of Onanong Thongmee et al. (Line 80-85)

2)    The target group of upstream is the Fresh market in Chiang Rai, which is the biggest fresh market in Chiang Rai, and it is a big source of raw material distribution. (Line 176-178)

3)    The target group of midstram: we have added the inclusion criteria for the midstream study in lines 224-228. A purposive sample size diagram for traditional Lanna restaurants has also been constructed and is now presented in Fig. 3. (Page 7)

4)    The target group of downstream, we collect from the 305 customers who have ever experienced traditional Lanna Cuisine. Moreover, G*Power is used for the sample calculation for the downstream operation. (Page 8)

5.2 *Line 94 and 209: The term E. coli and EMB agar should be written out in full initially before using the abbreviated form.*

**The revision:**

From this concern, we revise for the full inially as the before using the abbreviated form whereas the full name of *Escherichia coli* is illustrated at line 93, and the full name of Eosin Methylene Blue (EMB) agar is displayed at the line 230.

5.3 *Please carefully check the reference format in both reference lists and content following the journal format throughout the manuscript.*

**The revision:**

We check the reference format for both of intext citation and the list of reference according to the PLOs One Guildeline manual for the intext citation and reference writing.

5.4 *Equipment such as HPLC and column should include the manufacturer's brand, model, city, and country of origin*.

**The revision :**

From this recommendation, we add the comericial name of HPLC column as the SunFire ^TM^ C-18 column made in Ireland ( line 187-188, 190-191) , and the company of Himedia Co.Ltd, for the EMB agar (Line 230).

5.5 *All test kits should include the manufacturer's brand, city, and country of origin.*

**The revision:**

From this recommendation, we add the commercial name of the chemical test-kit called as the GPO, and this test-kit made in Thailand. This information is provided as the line 204-210.

*5.6 A statistical analysis section should be included to describe the experimental design, number of analytical replicates, and variance analysis methods, and the analytical tools employed*.

**The revision:**

Thank you for your important recommendation; we add the “cross-sectional design” for the study design as a line of 155. For the laboratory analysis, including the pesticide, chemical, and biological contamination, we have a triplicate for this analysis, as can be seen in lines 210-211 and 239 . In addition, we revise the data interpretation in the downstream using the IBM SPSS for analysis of the average, p-Value of the ANOVA test, the Levene’s test for homogeneity of variances ,and the effect size of factors from the Eta Square calculation, as can be seen in lines 283-287 and the supplementary material D.

5.7 *Figures 4 (3) and 5(4) should display error bars, and if statistical analysis indicates significant differences, these should be marked with appropriate symbols.*

**The revision:**

For this valuable recommendation, we create the error bar for fig. 4 as the 9’s page . However, in fig. 5 is the comparison of *E.coli* contamination from the pre-training and post-training at the midstream. Therefore, there is no evidence to support the error bars from SPSS associated with the chemical, and biological contamination. In addition, The p-Value at the downstream was changed to the appropriate symbols; especially the ANOVA score was then changed from p-Value = 0.00 to p-Value < 0.05 as can be seen in page 11.

.
