# Supplementary material for: The development of the “Laab Nuer Model” for food safety management in handling traditional Lanna cuisine in Thailand
Source: PLoS One. 2025 Sep 26;20(9):e0331933. doi: 10.1371/journal.pone.0331933 (PMC12469109; doi:10.1371/journal.pone.0331933)
Supplement: Attachment — Submitted filename: Reviewer comments-Plosone.pdf [file pone.0331933.s009.pdf]

Research Topic: The development of the "Laab Nuer Model" for food safety management in handling traditional Lanna cuisine at Thailand

Reviewer comments:
In my opinion, the revised manuscript entitled 'The development of the Laab Nuer Model for food safety management in handling traditional Lanna cuisine at Thailand' is quite comprehensive. However, it is necessary to revise the text further before it can be published.

1. As I cannot see whether the authors have comprehensively addressed the previous reviewer's comments, I have a question about this revised version: what is the rationale for determining this sample size?

2. Line 94 and 209: The term *E. coli* and EMB agar should be written out in full initially before using the abbreviated form.

3. Please carefully check the reference format in both reference lists and content following the journal format throughout the manuscript.

4. Equipment such as HPLC and column should include the manufacturer's brand, model, city, and country of origin.

5. All test kits should include the manufacturer's brand, city, and country of origin.

6. A statistical analysis section should be included to describe the experimental design, number of analytical replicates, and variance analysis methods, and the analytical tools employed.

7. Figures 3 and 4 should display error bars, and if statistical analysis indicates significant differences, these should be marked with appropriate symbols.
